# Genetic Adaptations, Biases, and Evolutionary Analysis of Canine Distemper Virus Asia-4 Lineage in a Fatal Outbreak of Wild-Caught Civets in Thailand

**DOI:** 10.3390/v12040361

**Published:** 2020-03-26

**Authors:** Chutchai Piewbang, Jira Chansaenroj, Piyaporn Kongmakee, Wijit Banlunara, Yong Poovorawan, Somporn Techangamsuwan

**Affiliations:** 1 Department of Pathology, Faculty of Veterinary Science, Chulalongkorn University, Bangkok 10330, Thailand; alkaline_eart@hotmail.com (C.P.); wijit.k@chula.ac.th (W.B.); 2 Animal Virome and Diagnostic Development Research Group, Faculty of Veterinary Science, Chulalongkorn University, Bangkok 10330, Thailand; 3 Center of Excellence in Clinical Virology, Faculty of Medicine, Chulalongkorn University, Bangkok 10330, Thailand; job151@hotmail.com (J.C.); yong.p@chula.ac.th (Y.P.); 4 The Zoological Park Organization under The Royal Patronage of H.M. The King, Bangkok 10800, Thailand; piyaporn.kmk@gmail.com

**Keywords:** Asia-4, canine distemper, civet, codon bias, evolutionary analysis

## Abstract

Canine morbillivirus (CDV) is a serious pathogen that can cause fatal systemic disease in a wide range of domestic and wildlife carnivores. Outbreaks of CDV in wildlife species lead to questions regarding the dispersal of the CDV origin. In the present study, we identified a fatal CDV outbreak in caged wild-caught civets in Thailand. Full-length genetic analysis revealed that CDV from the Asia-4 lineage served as the likely causative agent, which was supported by the viral localization in tissues. Evolutionary analysis based on the CDV hemagglutinin (H) gene revealed that the present civet CDV has co-evolved with CDV strains in dogs in Thailand since about 2014. The codon usage pattern of the CDV H gene revealed that the CDV genome has a selective bias of an A/U-ended codon preference. Furthermore, the codon usage pattern of the CDV Asia-4 strain from potential hosts revealed that the usage pattern was related more to the codon usage of civets than of dogs. This finding may indicate the possibility that the discovered CDV had initially adapted its virulence to infect civets. Therefore, the CDV Asia-4 strain might pose a potential risk to civets. Further epidemiological, evolutionary, and codon usage pattern analyses of other CDV-susceptible hosts are required.

## 1. Introduction

Canine morbillivirus, previously known as canine distemper virus (CDV), has caused epizootics and endemics of devastating diseases worldwide [1]. The CDV belongs to the *Morbillivirus* genus, *Paramyxoviridae* family, and contains a 15,690 bp negative-sense single-stranded RNA genome [2]. Similar to other paramyxoviruses, such as the measles virus (MeV) in humans, phocine morbillivirus (phocid distemper virus) in a large group of pinniped and marine mammals, and the rinderpest virus (RPV), the CDV possesses non-overlapping, unsegmented transcriptional RNA which encodes six core structural proteins [3,4,5]. These are the fusion (F), nucleocapsid, phospho-, large, matrix, and hemagglutinin (H) proteins, plus two minor structural proteins (C and V). The H protein is the most hypervariable region in the CDV genome [6].

Clinical presentations and pathogenesis of CDV-infected animals are consistent with respect to the variable degrees of morbidity and mortality among the infected species [7]. Infections between intra- and inter-species have been reported with evidence of transmission via aerosols [6] and either direct contact with infected secretion, blood, saliva, and urine or indirect contact with fomites. In addition, predation is also considered a transmission mode for CDV [8]. During infection, the CDV H protein initiates the infection by attaching to the signaling lymphocyte activating molecule (SLAM) receptor of immune cells [9,10] and nectin-4 receptors in various epithelial cells of the hosts [11,12,13]. The F protein then plays a crucial role in viral fusion on the host cellular membrane after H protein binding to the host cell [14,15]. After CDV infects the local lymphoid cells, the virus spreads to distant lymphatic tissues where T-cell and B-cell lymphocytes proliferate. The virus then infects other lymphocyte populations and epithelial cells, resulting in immunosuppression and systemic infection sequelae [16,17,18].

Among the CDV lineages, the *H* gene has shown the highest genomic variability and its action is potentially associated with viral tropism [19]. Thus, mutations in the *H* gene associated with viral binding entry sites (via either SLAM or nectin-4 cellular receptors) lead to fitness compensations between the virus and hosts and/or drive the evolution of CDV traits [20,21,22]. This scenario may result in the adaptation of CDV to be able to infect different hosts. Due to the global distribution of the CDV, phylogenetic genotyping of this virus has been based on the *H* gene sequence and segregated it into several major clades: America-1, America-2, Asia-1, Asia-2, Africa-1, Africa-2, European wildlife, Arctic, and Europe-1/SouthAmerica-1 [6], while the latest clades have recently been described as America-3 to -5, Asia-3 to -5, South America-2, and South America-3 [23,24]. In addition, the Caspian clade was recently isolated as the cause of a mass die-off of Caspian seals since 1988 [25].

Unlike the MeV, which has single host susceptibility, the CDV-associated diseases have been described not only in domestic dogs but have also been reported in endemics across several species, including wild canids, large felids, phocids, and procyonids, in addition to sporadic epizootics documented in ailurids, ursids, hyaenids, mustelids, and viverrids [7,26,27,28,29,30,31,32]. Moreover, uncommon CDV outbreaks have been reported in large-scale macaques [33], captive marmots [34], and an individual case of a collared anteater [35]. CDV-induced disease can vary in severity from subclinical to fatal outcomes but is commonly characterized by respiratory, intestinal, neurological, and hematological abnormalities with some pathology of integuments [36].

Understanding CDV evolution would likely offer important insights into the potential future threats of cross-species infections, which is important not only in disease control of domestic animals but also for the development of a disease prevention strategy in susceptible wildlife species. In the present study, we identified, characterized, and investigated the evolutionary pattern and codon usage bias of the CDV strain responsible for a fatal outbreak of wild-caught Asian palm civets raised in captivity for perfume production in Thailand. Using the generated complete CDV genome sequence to identify the viral protein in the intestinal tissues of a moribund civet suggested the CDV Asia-4 strain served as the causative agent circulating in the outbreak. Evolutionary analysis and synonymous codon usage analysis revealed that the CDV Asia-4, which diverged from the domestic dog’s origin, has mutated and adapted to infect civets.

## 2. Materials and Methods

### 2.1. Animals and Sample Collection

In January 2017, eight wild-caught Asian palm civets (*Paradoxurus hermaphrodites*) that were being raised in captivity for perfume production in Kanchanaburi Province, Thailand, were found dead. Due to the availability of the animals and local regulation permits related to carcass movement, on-site necropsy was performed only on the last dead civet by responsible veterinarians. Unfortunately, only the lung and intestinal tissues of this necropsied civet were collected and submitted for further histopathology, immunohistochemistry (IHC), and molecular analysis at the Department of Pathology, Faculty of Veterinary Science, Chulalongkorn University. Two fecal samples from the other moribund civets were also collected, but the fresh specimens of the other dead civets were unfortunately destroyed, and samples of free-roaming dogs or other animals were not available, and were therefore not included in this study.

### 2.2. Postmortem Examination

Postmortem examination was performed and recorded on a dead civet by responsible veterinarians. For the histology, the lung and intestinal samples were immersed in 10% (*v/v*) neutral buffered formalin, processed, and further embedded in paraffin. Tissue sections were then cut at a thickness of 4 μm and were stained with hematoxylin and eosin (HE) using routine procedures prior to investigation under light microscopy. The fresh intestinal tissue derived from the necropsied civet and the two fecal samples obtained from two other moribund civets were stored at −80 °C for molecular study.

### 2.3. General Virologic Testing

The fresh tissue and fecal samples were individually homogenized in 1% (*v/v*) phosphate-buffered saline (PBS), and the total viral nucleic acid was extracted using a commercial viral nucleic acid extraction II kit (Geneaid, Taipei, Taiwan) following the manufacturer’s recommendation. The extracted nucleic acids were then qualified and quantified spectrophotometrically using the A_260_/A_280_ absorbance ratio (NanoDrop, Thermo Scientific™, MA, USA). The extracted DNA/RNA was further subjected to polymerase chain reaction (PCR) analysis to screen for viruses using several pan-virologic-family PCR testing panels, including the detection of the herpesvirus [37], paramyxo-pneumovirus (PMX) [38,39], parvovirus [40], and coronavirus [41]. Subsequently, the PCR amplicons were visualized using a QIAxcel capillary electrophoresis platform and a Qiaxcel DNA screening kit (Qiagen, Hilden, Germany). Analysis of the DNA fragment under each PCR condition and DNA size marker used in this run was performed as previously described [42]. The positive samples of the PMX-PCR were further used to differentiate the virus using the individual PCR targeting *H* gene of CDV [23], nucleoprotein of the parainfluenza virus [43], and large polymerase gene of the pneumovirus [42]. The positive amplicons in each reaction were purified using a NucleoSpin^®^ Extract II kit (Macherey-Nagel, Düren, Germany) and were then commercially Sanger sequenced at Macrogen Inc. (Seoul, South Korea).

### 2.4. Full-Length Genomic Characterization of the Civet CDV

Since the preliminary results from the general virologic screening revealed that all the extracted intestinal and two fecal swab samples were positive for the pan-paramyxovirus PCR detection and showed 100% sequence identity of the *H* gene among tested samples, only the extracted viral nucleic acids from the intestinal tissue of a necropsied civet were subjected to full-length genomic characterization using one-step reverse-transcription (RT)-PCR. Briefly, the RT-PCR was performed using a Qiagen One-step RT-PCR kit (Qiagen, Hilden, Germany) with the oligonucleotide primer set for whole genome sequencing and thermocycling conditions as previously reported [23]. The PCR amplicons were resolved in 2% (*w/v*) agarose gel electrophoresis and visualized under a UV light illuminator. Subsequently, the positive PCR amplicons were further purified and extracted using a Monarch DNA Gel extraction kit (New England Biolab, Frankfurt, Germany) prior to submission for commercial Sanger sequencing as stated above.

### 2.5. Genome, Phylogeny, and Recombination Analyses of the Full-Length Civet CDV Genome

All civet CDV sequences derived from each PCR reaction were aligned and used to construct the consensus whole genome using Bioedit. The complete genome sequence of civet CDV, named herein CDV-CP8 TH/2017 after this outbreak, was then aligned with previously published CDV sequences retrieved from GenBank using the MAFFT online alignment server version 7 (https://mafft.cbrc.jp/alignment/server/) and MEGA 7 software package version 7.0. Deduced amino acid sequences were obtained by in silico translation from the alignment of the full-length civet CDV genome and allegorized with other published CDV sequences.

In addition, the alignments were used as a template for phylogenetic analysis. The phylogenetic tree was constructed using the maximum likelihood (ML) method with a general time reversible (GTR) model as the best-fit model nucleotide substitution according to the Bayesian information criterion. The phylogenetic tree was bootstrapped with 1000 replicates, conducted in the MEGA 7 software package. Nucleotide pairwise distances of the civet CDV complete genome were estimated using MEGA 7. Further analysis of the deduced amino acids of the complete H gene was performed by comparison with the 241 available CDV sequences from various hosts in GenBank.

In order to find out the likely origins of the CDV in this outbreak and to identify any potential recombination in CDV-CP8 TH/2017, the CDV alignments were subjected to recombination analysis using the statistically integrated Recombination Detection Program (RDP) software package version Beta 4.94. The recombination breakpoints were analyzed by several methods including RDP, Boostscan, GeneCoV, MaxChi, Chimera, SIScan, and 3Seq with a cut-off *p*-value at 0.01. The other general settings of the program were previously described [23,44].

### 2.6. Evolutionary Analysis of the CDV H Gene

In order to further identify the origin of civet CDV-CP8 TH/2017, the 241 retrieved CDV *H* gene sequences were used to perform an evolutionary analysis with the potential civet CDV-CP8 TH/2017 using the Bayesian Markov Chain Monte Carlo model implemented in BEAST v2.4.8 [45]. The jModelTest [46] was performed to identify the best fitting nucleotide substitution model for the multiple alignment sequences. The best-fit substitution model under a log-normal relax and strict clock model at constant population sizes as priors were implemented to account for varied evolutionary rates among lineages. The coalescent Bayesian skyline tree prior and empirical base frequencies were conducted under the best-fit clock model and run for 100 million chains, sampling every 10,000th generation, with the first 10% being discarded as burn-in. The convergence of parameters was confirmed by calculating the effective sample size using the TRACER program v.1.7.0 [47]. The maximum clade credibility (MCC) trees were annotated using TreeAnnotator v.1.8.3 [45]. Phylogenetic trees with estimated divergence, variable timeline, posterior probability, and 95% highest posterior density (HPD) were generated and displayed using FigTree v.1.4.3 [47].

### 2.7. Codon Usage Analysis of the Asian CDV H Gene

The CDV *H* gene has been found to have the highest divergence among the CDV genomes, and so it has been used for strain classification. Furthermore, most studies of CDV isolates have focused on the mutations in the *H* gene and found that only Asian strains of CDV have been reported in wildlife species on the Asian continent. Thus, the available various Asian CDV *H* genes were further analyzed in this study. To indicate the potential evolutionary changes of CDV associated with the ability to infect the civet in this outbreak, an estimation of the codon usage bias pattern was studied in terms of both sequence composition and the relative synonymous codon usage (RSCU).

#### 2.7.1. Sequence Information and Recombination Sequence Removal

Due to the fact that genetic recombination can alter any interpretation of codon usage bias, the recombinant CDV strains were excluded from this study. Thus, the exploration of codon usage bias was performed on the complete *H* gene of 31 CDV strains retrieved from the GenBank database (Supplementary Table S1, Supplementary Info).

#### 2.7.2. Nucleotide and Codon Usage Composition of CDVs

The sequence compositions of the coding H protein from various CDV lineages were analyzed in terms of (i) the nucleotide composition by percentages of A, T, C, and G using BioEdit, (ii) the GC nucleotide frequency at the first, second, and third positions of synonymous codons (GC1s, GC2s, and GC3s), and (iii) the specific codon composition at the third position (A3%, U3%, C3%, and G3%) which were all calculated using MEGA 7. A total of five non-biased codons, comprised of two codons designating only Met and Trp (ATG and UGG, respectively) and three stop codons (UAA, UAG, and UGA), were excluded from the analysis.

#### 2.7.3. Relative Synonymous Codon Usage (RSCU) of Asian CDVs and between CDV-Susceptible Host Species

To investigate the possible origin of the CDV-CP8 TH/2017 in this outbreak and to estimate the trend of preferable hosts for Asian CDV strains, which are endemic to the Asian continent, the RSCU analysis was applied to the complete *H* gene of 20 CDV Asian strains (Supplementary Table S1). The codon usage bias of the CDV was directly calculated using the RSCU values of a synonymous codon, referring the relative probability of its detected frequency to the expected frequency assuming that all potential codons were used equally. Thus, the RSCU values were calculated from Equation (1) [48,49]:

(1) $$RSCU=\frac{g_{ij}}{\sum_{j}^{n_{i}}g_{ij}}ni$$

where *g_ij_* is the occurrence number of the *i*_th_ amino acid that has n_i_ representing the number of synonymous codons encoding a specific amino acid. An RSCU value greater than 1.0 indicates a positive codon usage bias, whereas a value less than 1.0 represents a negative codon usage bias. Furthermore, RSCU values >1.6 indicate overrepresented codons, while values <0.6 suggest underrepresented codons, as described previously [50]. The RSCU values were implemented and calculated using MEGA 7.0.

Additionally, to identify the preference of the CDV *H* gene to the civet host compared with the natural host (dog), the RSCU values of Asian CDVs relative to CDV-susceptible host species (domestic dog; *Canins familiaris* and Asian Palm civet; *Paradoxurus hermaphroditus*) were calculated. The codon usage tables for *C. familiaris* and *P. hermaphroditus* were directly retrieved from the publicly available Codon Usage Database (www.kazusa.or.jp/codon). The evidence of shared codon preference between CDV genotypes and susceptible hosts was considered if the particular codon had the highest RSCU value in the hosts similar to the virus.

### 2.8. Detection of CDV in Civet Tissues by IHC Analysis

To further confirm the presence of CDV in tissue, the lung and intestinal formalin-fixed paraffin-embedded (FFPE) sections from the necropsied civet were tested for CDV by IHC staining using the chain polymer-conjugated method [26]. Briefly, the 4-µm FFPE sections were deparaffinized and rehydrated with xylene and a sequential decreasing ethanol gradient. The CDV antigen was retrieved by immersing the sections with distilled water followed by incubation in an autoclave at 121 °C for 5 min and then treatment with 3% (*v/v*) hydrogen peroxide in methanol for 5 min to block any endogenous peroxidase activity. Subsequently, the slides were washed with PBS three times and then incubated with a 1:200 dilution of mouse-monoclonal antibody against CDV’s envelope proteins F and H (Monotope; ViroStat, ME, USA) as primary antibodies, at 37 °C for 60 min. The slides were then incubated using the Dako REAL EnVision Detection System (Dako, Glostrup, Denmark), at 37 °C for 60 min as a secondary antibody, washed with PBS three times, and the positive antigen–antibody complex was then visualized by labeling with 3, 3′-diaminobenzidine tetrahydrochloride (DAB) and counterstaining with Mayer’s hematoxylin. Positive control slide was performed on brain tissue from a CDV-infected dog that was positive in the RT-PCR assay, while the Tris-HCl buffer instead of the primary antibody was used as a negative control.

### 2.9. Ethics Statement

The authors confirm that the ethical policies of the journal, as noted on the journal’s author guidelines page, have been adhered to, and the appropriate ethical review committee approval has been received. All experimental protocols were approved by the Chulalongkorn University Animal Care and Use Committee (No. 1631002). Date of approval on February 17th, 2016. All procedures were done in accordance with the relevant guidelines and regulations.

### 2.10. Data Availability Statement

The data that support the findings of this study are available from the corresponding author upon reasonable request.

## 3. Results

### 3.1. Animal Habitats and Potential History

All eight Asian Palm civets were originally caught from the frontier’s forest in the west of Thailand and the east of Myanmar’s border with unknown precise origin and actual catching date. The wild civets were individually kept in a bamboo cage, located in a poorly-restricted, uncontrolled area. Since the first moribund civet was found, the remaining civets continuously showed depression, severe dehydration, followed by watery diarrhea and hyperkeratosis at all footpads and nasal planum, and then died within a week of onset of the symptoms.

### 3.2. Pathological and General Virologic Findings

Upon necropsy of the dead civet, the carcass was found to be dehydrated with a poor body condition. Evidences of progressive watery diarrhea was found in terms of fecal staining at the perineum and thickening of the epidermis at the nasal planum and all footpads. The remarkable morphological findings of the internal organs were severe acute diffuse pulmonary congestion and edema, segmental catarrhal enteritis with a massive yellowish intraluminal content, and focal necrotizing hepatitis.

**Figure 1:**
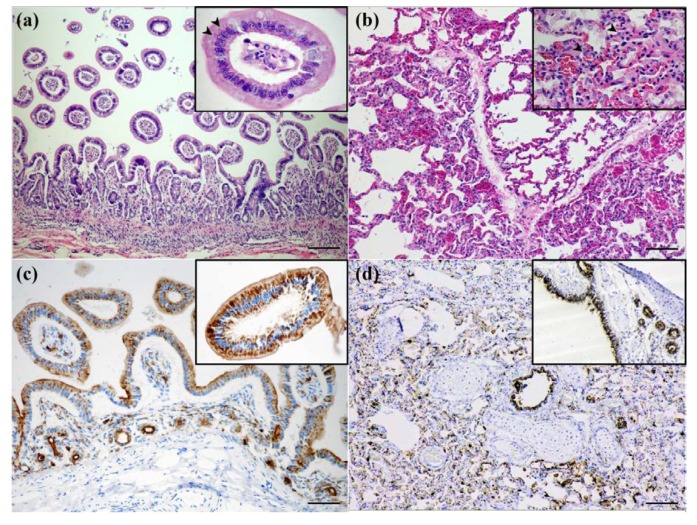
Civet tissue staining. (**a**,**b**); H&E staining showing (**a**) severe chronic lymphoplasmacytic enteritis with eosinophilic intranuclear and intracytoplasmic inclusion bodies that are frequently seen within villous epitheliums (inset, arrowheads) and (**b**) severe broncho-interstitial pneumonia with eosinophilic intracytoplasmic inclusion bodies that were randomly observed in pneumocyte-lining alveoli (inset, arrowheads). (**c**,**d**), IHC staining showing the immunopositive CDV antigen was diffusely observed within (**c**) cryptal and villous epitheliums (inset) and (**d**) in pulmonary and bronchial epitheliums (inset). Scale bar = 200 µm.

With respect to available samples and gross lesions of the intestinal and lung tissues, the histology revealed chronic enteritis with a mild to moderate infiltration of lymphocytes and plasma cells and a variable level of accumulation of tissue debris. The gut-associated lymphoid tissues were moderately hyperactive. Frequent eosinophilic intranuclear and intracytoplasmic inclusion bodies were observed within the villous epitheliums and infiltrated mononuclear inflammatory cells (Figure 1a).

The lung section revealed severe broncho-interstitial pneumonia, characterized by a mixed infiltration of inflammatory mononuclear cells and neutrophils (Figure 1b). Sloughed cellular debris and fibrin frequently occupied larger airways. Eosinophilic intracytoplasmic inclusion bodies were randomly observed in the bronchial epitheliums adjacent to the inflammation area.

The general virologic screening of the fresh fecal and intestinal samples derived from three civets revealed positive PCR reactions specific for paramyxoviruses, while they were negative for the other screening tests. This finding, therefore, indicated that CDV may be a possible etiological pathogen in this outbreak and prompted us to further characterize the complete genome sequence of the particular CDV circulating in these civets and to perform the specific-IHC assay for CDV on the lung and intestinal FFPE tissues of the moribund civet.

### 3.3. CDV Localization in Tissues

With respect to the limited samples of a single moribund civet, the CDV distribution in all the other tissues apart from the lung and intestine could not be determined. The IHC analysis of the lung and intestine revealed that the CDV-specific antigen was diffusely observed in the intestinal villous and cryptal epitheliums (Figure 1c), as well as within the cytoplasm of alveoli-lining pneumocytes, bronchial epitheliums, and sloughed cells, wherein the histological lesions were present (Figure 1d).

### 3.4. Genetic Characterization and Phylogenetic, Recombination, and Evolutionary Analyses of the Complete Genome of Civet CDV 

**Figure 2:**
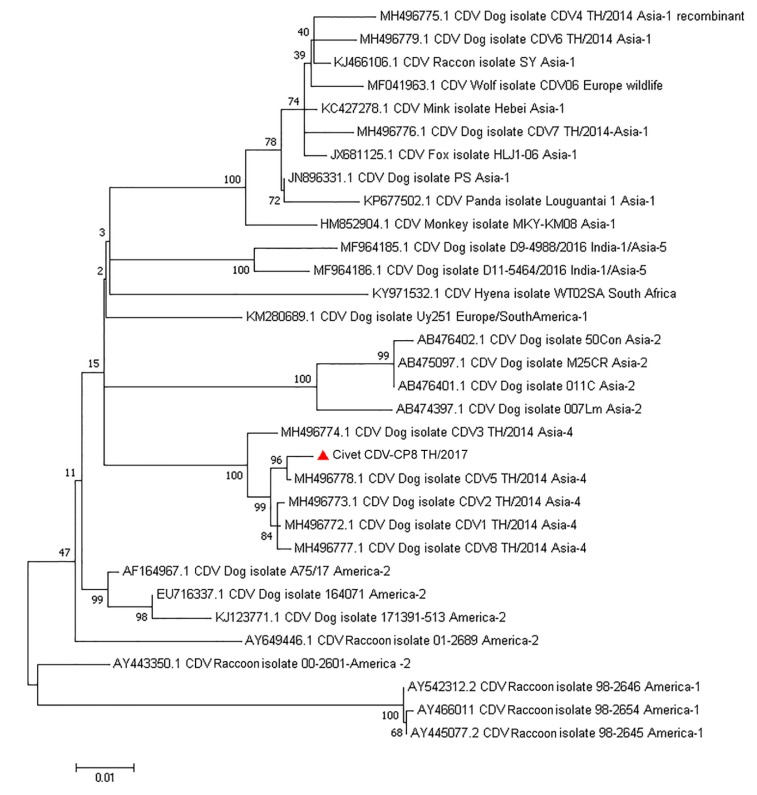
Phylogenetic analysis of the civet CDV strain in this study. Phylogenetic trees were based on the full-length CDV genomes, which revealed that the civet CDV-CP8 TH/2017 (▲) isolated in Thailand clustered with the CDV Asia-4 lineage. Bootstrapping results for 1000 replications with values greater than 50% are shown for each node. CDV (canine distemper virus).

Since there was 100% sequence identity of the CDV strains among the tested samples in the preliminary results of the pan-PCR, only the extracted nucleic acids derived from the intestinal sample of the necropsied civet were used for whole genome sequencing. The complete genome sequence of the circulating civet CDV (CDV-CP8 TH/2017; GenBank accession no. MT149210) contained 15,690 bp and encoded six core structural proteins. The pairwise nucleotide distances of CDV-CP8 TH/2017 ranged from 0.7–7% nucleotide differences among the various CDV stains, with the highest genetic similarity to the CDV Asia-4 isolate CDV-5 TH/2014 (MH496778). Phylogenic topology (ML tree) of the CDV *H* gene supported the finding of the pairwise distance analysis and indicated that the CDV-CP8 TH/201 was an Asia-4 strain (Figure 2). For the deduced amino acids of the CDV *H* gene that are potentially involved in binding to the host SLAM and nectin-4 receptors, the civet CDV revealed amino acid residues 530D, 540D, and 549H [26]. Analysis of the derived civet CDV sequence for genetic recombination using statistically-based programs, including RDP, Boostscan, GeneCoV, MaxChi, Chimera, SIScan, and 3Seq, revealed no evidence of genetic recombination in CDV-CP8 TH/2017.

**Figure 3:**
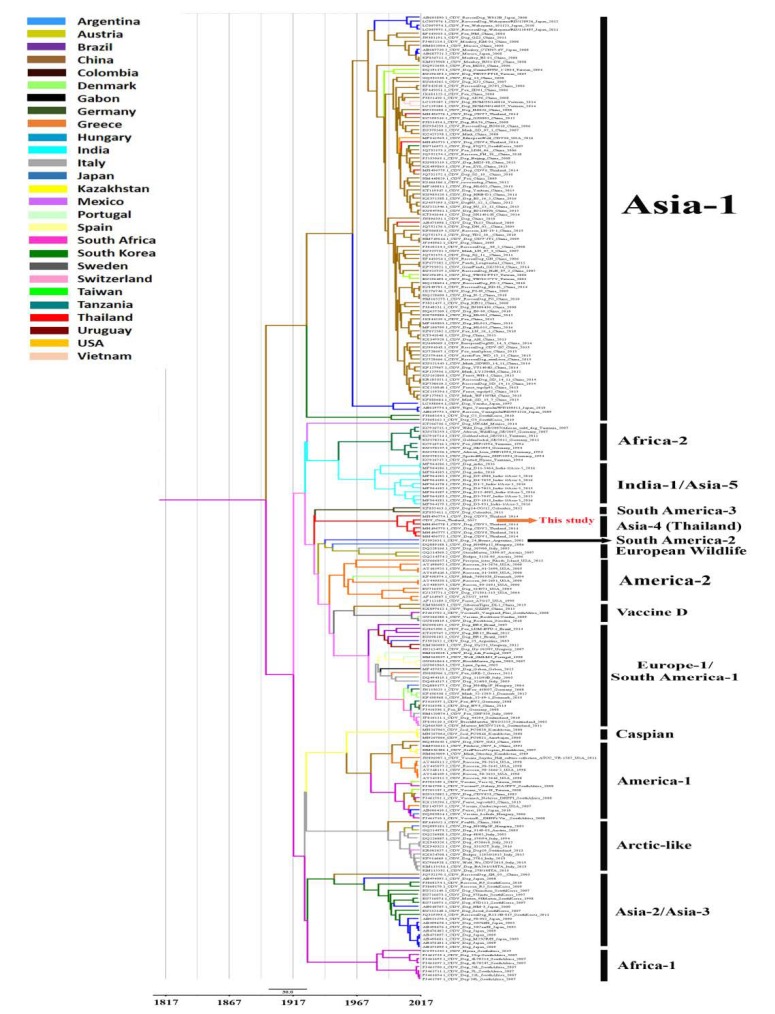
Maximum clade credibility (MCC) tree of 241 full-length H genes of CDVs available in the GenBank database for the 1975–2017 period. The MRCAs of these strains are indicated with their dates of existence. The phylogenetic branches are colored according to geographical locations in which the strains were detected.

To further identify the potential origin of the civet CDV-CP8 TH/2017 strain detected in this outbreak, the complete *H* gene sequence was compared with the previously published CDV genomes available in GenBank. The dataset of 241 CDV *H* gene sequences isolated from various animals in 27 countries between 1975 and 2017 was utilized to estimate the divergence variable timeline and evolutionary history of CDV. The suitable best-fit substitution model for these aligned sequences was the GTR model with a strict clock model. The overall evolutionary rate of the CDV *H* gene sequences was estimated at 3.87 × 10^−4^ substitutions/site/year (95% HPD: 2.96–4.65 × 10^−4^). The time to the most recent common ancestor (tMRCA) of the CDV cluster, which was clustered based on the previously reported clades [25], was estimated to be approximately 1817 (95% HPD:1802–1832). The tMRCA of the CDV Asia-4 cluster was estimated to have emerged in about 1927 (95% HPD:1912–1942), whereas the evolutionary tree revealed that the CDV sequence derived from civet was uniquely clustered in the Asia-4 lineage and co-circulated with CDV strains isolated from domestic dogs in Thailand since 2014 (Figure 3).

### 3.5. Frequent Nucleotide Composition and the RSCU of the Asian CDV H Gene

The demonstration of genome usage bias is frequently analyzed by estimating the nucleotide composition and codon usage bias in the highly variable region of the target organism’s genome. The codon usage bias is frequently used as a tool to investigate viral evolution. Thus, we estimated the nucleotide composition and RSCU bias of the CDV strains to identify any bias in the Asian CDV *H* gene.

Overall, A and U(T) were the most frequent mononucleotides observed in the *H* gene of CDV strains, accounting for 29.77 ± 0.23% and 27.70 ± 0.20%, respectively, while the G and C contents were less frequent, at 21.42 ± 0.19% and 21.11 ± 0.20%, respectively. As a result, the dinucleotide composition of AU(T) (28.73 ± 1.06%) was higher than that of GC, with average GC contents of 21.32 ± 4.38% and 21.15 ± 0.66% at the first and second and at the third positions (GC_12_ and GC_3_), respectively. Further analysis of the codon frequency at the first and second positions revealed that A_1_ and U_2_ were the most abundant synonymous codons, accounting for 30.68 ± 0.45% and 32.25 ± 0.20%, respectively, while the third position had A_3_ and U_3_ as the most abundant codons, at 29.64 ± 0.67% and 28.05 ± 0.56%, respectively (Supplementary Table S1; Supplementary Info).

Additionally, we analyzed the nucleotide compositions among the CDV Asian phylogenetic clades including civet CDV-CP8 TH/2017, which revealed similar average nucleotide compositions, with A and U being more abundant in the five CDV Asian clades (Supplementary Table S2). The presence of the dissimilar nucleotide composition in the *H* gene among the different CDVs revealed that codon usage bias occurred.

To estimate the codon usage pattern for the *H* gene of the Asian CDVs, the RSCU was evaluated. Patterns of preferable synonymous codon usages were found in the Asian CDV strains; 14 were A/U-ended codons with eight terminated by A, while the numbers of U-, G-, and C-ended codons were 6, 3, and 1, respectively, (Table 1). Out of 18 optional synonymous codons, six were overrepresented (RSCU values over 1.6), of which five were A-ended codons (UCA, ACA, GCA, AGA, and GGA), and there were no underrepresented synonymous codons (RSCU values less than 0.6).

Further analysis of the synonymous codon usage tendencies among the Asian CDV strains revealed A/U-ended codons were more common synonymous codons than C/G-ended codons, showing that A-ended codons were the most preferable codon usage. The preferably synonymous codon usage was consistent with the occurrence among Asian CDV strains, except for the optional codons for Phe (F), Leu (L), Ile (I), Pro (P), His (H), Gln (Q), Lys (K), and Glu (E) (Table 1). Focusing on the codons encoding His, there was no synonymous codon usage bias in CDV Asia-1, whereas the preferred codon was CAU in the CDV Asia-2 and -4, and CAC in the CDV Asia-3 and -5 strains.

### 3.6. Relative Synonymous Codon Usage between Asian CDV and Selective Hosts

We further explored the relationship between CDV Asian strains and their natural host (dogs) as well as the recent host (civets) found in this study. The 18 most abundant synonymous codons were unharmonious between the CDV Asian strains and their natural and emerging hosts. However, four codons were preferentially used in all the Asian CDV strains and their hosts (UAU, AAU, GAU, and GGA), while two preferential codons found in all Asian CDV strains (ACA and AGA) were specific to civets. Surprisingly, we found that UAU, encoding for Tyr, was the most preferred codon between the CDV Asian strains and hosts, whereas CAU (encoding for His) was the preferable codon between hosts and the CDV Asian-2 and -4 strains. Focusing on the RSCU pattern of the CDV Asia-4 with its natural host (dog) and the civet, this finding indicated the RSCU patterns of the CDV Asia-4 were more closely related to the civet’s pattern at codons encoding for Pro (P), Thr (T), and Arg (R) than the patterns observed in dogs (Table 1).

## 4. Discussion

The CDV is known as a primary fatal pathogen that causes serious systemic diseases in susceptible carnivores. Apart from canids, viverrids have been recognized as a vulnerable host for CDV infection [7]. Since the CDV was first detected at a large-scale in susceptible hosts, viral outbreaks in civets have been documented in an individual case of a genet (*Gennetta gennetta*) in Spain in 1999 [51], an epizootic occurrence in captive masked palm civets in Japan during the 2005–2007 period [52], and at a civet farm that contained a colony of masked palm civet (*Paguma larvata*), Asian palm civet (*Paradoxurus hermaphroditus*), and small Indian civet (*Viverricula indica*) in Thailand in 2011 [26]. At the time, the CDV Asia-1 strain, which was recognized as a common CDV strain isolated from domestic dogs in Thailand [53], served as the cause of that outbreak. The potential horizontal transmission mode between interactions from roaming dogs and infected civets remains equivocal and requires clarification [26].

In this study, we reported the CDV Asia-4 lineage associated with an outbreak in captive wild-caught civets in Thailand by performing full-length genome characterization, IHC, and phylogenetic and evolutionary analyses. These results indicated the expansion of the CDV lineage, apart from Asia-1, spreading with fatal outcome in viverrid species.

The CDV Asia-4 lineage was first discovered in domestic dogs in Thailand in 2013 [23] and was subsequently identified in domestic dogs in China [54] and Russia [55]. Evolutionary analysis of the *H* gene indicated that the CDV Asia-4 lineage emerged and diverged from the North America-2 lineage in about 1924 [23]. Previous studies of the CDV Asia-4 infected host range were limited due to the detection being performed only in domestic dogs with no report in other wildlife species. Thus, we confirmed that the CDV Asia-4 lineage has potentially infected civet species and presented the clinical signs through pathological findings that confirmed that CDV was localized to areas in the lung and intestinal tissues of perturbed histology in the civet, similar to that found in CDV-infected dogs. Due to the limitation of available tissue, the viral distributions in other organs associated with pathological changes caused by CDV Asia-4 in civets could not be determined.

Dispersal of CDV via horizontal transmission may result from the interactions between domestic dogs and other susceptible hosts (i.e., domestic dogs, feral canids, and other wildlife species) and may lead to massive mortality in new host species [56] and vice versa, i.e., that the virus may spillback from wildlife reservoirs to domesticated dogs [57]. Annihilation of the original habitat of wildlife hosts probably forces animals to expand their residence to urban areas and so can result in an increased chance of horizontal transmission of pathogens, including CDV. Furthermore, unlike the MeV that has infected single host species, the host range of CDV is rather diverse and causes disease in a variety of different wildlife species.

In this study, the Asia-4 isolate of CDV isolated from three moribund civets shared 100% nucleotide similarity of the *H* gene to each other, and the full-length genome characterization of one of the CDV strains (CDV-CP8 TH/2017) isolated from the moribund civet had the highest nucleotide similarity to the CDV Asia-4 isolate CDV-5 TH/2014, which was previously isolated from a domestic dog in Thailand. This finding questions the origin of the civet CDV-CP8 TH/2017 of this outbreak. Since it was not possible to collect samples from surrounding animals, we investigated the possible origin of this virus using various genetic analytic algorithms to analyze the CDV *H* gene, which is associated with the viral binding process to host SLAM and nectin-4 receptors and the role of disease emergence in novel host species. The evolutionary analysis of the CDV *H* gene in this study suggested that this virus has co-evolved and clustered with the CDV strains isolated from dogs in Thailand in 2014. Thus, the possible transmission of CDV in this outbreak has domestic dogs as a potential origin. However, this conclusion remains tentative due to the unavailability of samples from other animals in the area, including dogs.

Understanding CDV evolution offers important insights into the potential future threats of cross-species infection. Estimation of the codon usage pattern is a direct way to indicate evolutionary changes in CDV that may increase its potential infection of other susceptible hosts [49]. Although each amino acid is translated from a single codon (nucleotide triplet), each amino acid can be encoded by different codons, so-called synonymous codons. Importantly, the frequency of use of synonymous codons is not random but is biased, known as “codon usage bias”, and varies between divergent species. Thus, codon usage bias is often observed in viruses and other organisms.

Analysis of the codon usage bias of Asian CDVs was compared among the Asian CDV strains by estimating the nucleotide composition through the RSCU. The A/U-ended codons as well as mono-, di-, and triple-codons were more frequent in all CDV Asian strains, emphasizing that the A/U-preferred codon governed the selective pressure on the Asian CDV *H* gene. This phenomenon has likely played an important role in the Asian CDV evolution. While dissimilar nucleotide compositions were preferred among CDV Asian strains, this finding emphasized that codon usage bias occurred in the CDVs and may have affected CDV evolution.

Mutation pressure, the external environment, selective transcription, and natural selection are listed as factors that determine codon usage bias. As with other RNA viruses [58,59,60,61], CDV depends on the host susceptibility with respect to survival and transmission. Thus, the CDV codon usage pattern may interact with the virus’ ability to infect, adapt, and escape from the host immune responses. Whether different CDV lineages are associated with distinct evolutionary changes, and if these changes are influenced by the host, remain unknown.

In this study, the RSCU analysis of the Asian CDVs and their natural hosts (dogs) and the new host (civet), revealed that the codon usage patterns of CDV Asia-4 were more similar to the pattern of the civets than that of dogs, which indicates the possibility of the discovered CDV-CP8 TH/2017, or its progenitors having adapted its virulence to infect the civets [62]. Furthermore, we also observed that there was no synonymous codon usage bias in the His-encoded codons (UAU or CAU) for the CDV Asia-1 lineage, while there was a codon usage bias pattern observed in these codons in the CDV Asia-4.

Of note, the preferable codon of UAU or CAU altered an encoding amino acid at position 549 in the *H* gene, from Tyr (Y) to His (H), which was found as 549H in the civet CDV-CP8 TH/2017. This site is important for binding to the SLAM receptor and is specific for host susceptibility [2,9,10,63]. Thus, the codon bias pattern at this site may be associated with the expansion of the host susceptibility and may extrapolate to the infectivity of CDV Asia-4 in civets.

Furthermore, in contrast to the other Asia-4 CDV strains, the RSCU pattern of CDV Asia-1 was more similar to the RSCU pattern of dogs than to that of civets. This finding may support the previous report of a CDV outbreak at a civet farm in Thailand that indicated the CDV Asia-1 was the origin of the outbreak and could have originated from free-roaming dogs. However, further investigation of CDV in wildlife species will allow more conclusive results.

In conclusion, this study identified the CDV Asia-4 lineage associated with a recent fatal outbreak in caged wild-caught civets in Thailand as the first report. The evolutionary and synonymous codon usage bias analysis supports the CDV origin, which arose and adapted from dogs as its natural host. This study will motivate further studies into the large-scale CDV surveillance in both domesticated dogs and other wildlife species that may be fruitful for disease surveillance and control. Expansion of CDV susceptible hosts should be taken into consideration.

## Figures and Tables

**Table 1:** The RSCU of CDV Asian strains and their potential hosts.

Amino Acid	Codon	CDV Strain						Potential Host	
		All	Asia-1	Asia-2	Asia-3	Asia-4	Asia-5	*C. familiaris*	*P. hermaphroditus*
**Phe (F)**	UUU	0.98	1.04	0.98	0.92	0.85	0.98	**1.09**	**1.06**
	UUC	**1.02**	0.96	**1.02**	**1.08**	**1.15**	**1.02**	0.91	0.94
Leu (L)	UUA	0.98	0.98	1.2	1.22	0.9	0.94	1.32	**1.78**
	UUG	**1.69**	**1.69**	**1.37**	1.13	**1.87**	**1.64**	0.51	0.4
	CUU	0.87	0.81	0.88	0.94	0.89	0.89	1.22	1.04
	CUC	0.65	0.69	0.67	0.56	0.47	0.66	1.01	0.74
	CUA	0.91	0.97	0.79	0.84	1.02	0.94	**1.42**	1.66
	CUG	0.91	0.86	1.09	**1.31**	0.85	0.94	0.51	0.37
Ile (I)	AUU	1.03	1.01	**1.06**	**1.08**	**1.12**	**0.98**	**1.05**	1.17
	AUC	0.88	0.8	0.94	0.96	0.86	0.91	0.94	0.65
	AUA	**1.09**	**1.19**	0.99	0.96	1.02	1.11	1.01	**1.19**
Val (V)	GUU	0.71	0.64	0.44	0.27	0.65	0.63	1.12	1.17
	GUC	1.02	0.99	1.05	0.98	1.1	1.08	0.57	0.62
	GUA	0.98	0.99	1.1	1.24	1.03	1.03	**1.67**	**1.81**
	GUG	**1.29**	**1.37**	**1.41**	**1.51**	**1.23**	**1.26**	0.64	0.4
Ser (S)	UCU	0.83	0.93	0.75	0.58	0.72	0.94	**1.35**	**1.61**
	UCC	1.36	1.47	1.49	1.58	1.4	1.18	1.04	1.1
	UCA	**2.02**	**2.03**	**1.85**	**1.85**	**2.02**	**2**	1.27	1.37
	UCG	0.37	0.27	0.6	0.58	0.42	0.35	0.39	0.28
	AGU	0.7	0.49	0.81	0.81	0.74	0.88	0.91	0.72
	AGC	0.72	0.81	0.51	0.58	0.7	0.65	1.05	0.92
Pro (P)	CCU	**1.39**	**1.59**	1.21	**1.44**	1.31	1.13	**1.41**	1.3
	CCC	0.81	0.77	0.85	0.85	0.87	0.81	1.24	1.05
	CCA	1.23	0.92	**1.24**	1.05	**1.33**	**1.63**	0.92	**1.28**
	CCG	0.58	0.71	0.7	0.66	0.48	0.44	0.43	0.37
Thr (T)	ACU	1.06	1.18	0.89	0.86	1.12	0.9	1.35	1.18
	ACC	0.84	0.79	0.99	0.97	0.73	0.9	1.06	0.91
	ACA	**1.74**	**1.82**	**1.92**	**1.84**	**1.63**	**1.7**	1.16	**1.54**
	ACG	0.36	0.21	0.2	0.32	0.51	0.5	0.43	0.38
Ala (A)	GCU	1.22	1.14	1.17	1.28	1.51	1.25	1.07	1.16
	GCC	0.83	0.99	0.6	0.64	0.67	0.86	**1.27**	**1.36**
	GCA	**1.68**	**1.68**	**1.92**	**1.6**	**1.65**	**1.49**	1.18	1.32
	GCG	0.27	0.19	0.3	0.48	0.17	0.39	0.48	0.16
Tyr (Y)	*UAU*	**1.4**	**1.49**	**1.18**	**1.16**	**1.48**	**1.53**	**1.15**	**1.17**
	UAC	0.6	0.51	0.82	0.84	0.52	0.47	0.85	0.83
His (H) *	CAU	**1.02**	**1**	**1.22**	0.94	**1.05**	0.96	**1.2**	**1.03**
	CAC	0.98	**1**	0.78	**1.06**	0.95	**1.04**	0.8	0.97
Gln (Q)	CAA	**1.53**	**1.51**	**1.43**	0.9	**1.54**	**1.47**	**1.25**	**1.52**
	CAG	0.47	0.49	0.57	**1.1**	0.46	0.53	0.75	0.48
Asn (N)	AAU	**1.14**	**1.01**	**1.35**	**1.43**	**1.1**	**1.14**	**1.18**	**1.28**
	AAC	0.86	0.99	0.65	0.57	0.9	0.86	0.82	0.72
Lys (K)	AAA	**1.28**	**1.28**	**1.31**	0.84	**1.32**	**1.07**	**1.37**	**1.42**
	AAG	0.72	0.72	0.69	1.16	0.68	0.93	0.63	0.58
Asp (D)	GAU	**1.22**	**1.19**	**1.24**	**1.18**	**1.15**	**1.24**	**1.13**	**1.15**
	GAC	0.78	0.81	0.76	0.82	0.85	0.76	0.87	0.85
Glu (E)	GAA	0.75	0.68	0.85	**1.11**	0.79	0.87	**1.17**	**1.35**
	GAG	**1.25**	**1.32**	**1.15**	0.89	**1.21**	**1.13**	0.83	0.65
Cys (C)	UGU	**1.45**	**1.5**	**1.46**	**1.43**	**1.5**	**1.13**	0.89	0.92
	UGC	0.55	0.5	0.54	0.57	0.5	0.87	**1.11**	**1.08**
Arg (R)	CGU	0.58	0.57	0.57	0.46	0.6	0.59	1.17	0.78
	CGC	0.22	0.2	0.33	0.46	0.2	0.3	0.92	0.69
	CGA	0.56	0.64	0.33	1.08	0.6	0.59	0.71	1.18
	CGG	1.02	1.14	0.94	0.77	1	1.08	0.48	0.46
	AGA	**2.69**	**2.77**	**2.79**	**2**	**2.56**	**2.75**	1.29	**1.47**
	AGG	0.92	0.69	1.04	1.23	1.04	0.69	**1.42**	1.41
Gly (G)	GGU	0.94	0.93	0.92	1.1	0.89	0.94	1.02	1.07
	GGC	0.42	0.29	0.55	0.28	0.45	0.41	1.05	0.73
	GGA	**1.71**	**1.7**	**1.87**	**1.79**	**1.77**	**1.88**	**1.27**	**1.68**
	GGG	0.92	1.07	0.66	0.83	0.89	0.76	0.66	0.52

* Potential CDV *H* gene amino acid associated with the binding to the host SLAM/Nectin-4 receptor. Potentially preferred CDV codons, CDV strains, and potential hosts are displayed in bold.

## Supplementary Materials

The following are available online at https://www.mdpi.com/1999-4915/12/4/361/s1, Supplementary Table S1: The detailed information of the CDVs used in this study. Supplementary. Table S2: Average nucleotide compositions of CDV Asian strains Supplementary Info. Nucleotide compositions of the various CDV strains.
