# Supplementary material for: Genetic Adaptations, Biases, and Evolutionary Analysis of Canine Distemper Virus Asia-4 Lineage in a Fatal Outbreak of Wild-Caught Civets in Thailand
Source: Viruses. 2020 Mar 26;12(4):361. doi: 10.3390/v12040361 (PMC7232145; doi:10.3390/v12040361)
Supplement: Supplementary file 1 [file viruses-12-00361-s001.zip › viruses-751412-for conversion-suppl_/Supplementary Table S1_Civet-CDV_Viruses-Somporn.pdf]

Supplementary Table S1. The detailed information of the CDVs used in this study.

Hosts	Isolate name	lineage	Accession Nos.
**Raccoon**	SY	Asia-1	KJ466106.1
**Dog**	CDV6 TH/2014	Asia-1	MH496779.1
**Mink**	Hebei	Asia-1	KC427278.1
**Dog**	PS	Asia-1	JN896331.1
**Dog**	CDV7 TH/2014	Asia-1	MH496776.1
**Fox**	HLJ1-06	Asia-1	JX681125.1
**Panda**	Louguantai 1	Asia-1	KP677502.1
**Monkey**	MKY-KM08	Asia-1	HM852904.1
**Dog**	CDV1 TH/2014	Asia-4	MH496772.1
**Dog**	CDV2 TH/2014	Asia-4	MH496773.1
**Dog**	CDV8 TH/2014	Asia-4	MH496777.1
**Dog**	CDV5 TH/2014	Asia-4	MH496778.1
**Dog**	CDV3 TH/2014	Asia-4	MH496774.1
**Dog**	007Lm	Asia-2	AB474397.1
**Dog**	50Con	Asia-2	AB476402.1
**Dog**	011C	Asia-2	AB476401.1
**Dog**	M25CR	Asia-2	AB475097.1
**Dog**	D9-4988/2016	India-1/Asia-5	MF964185.1
**Dog**	D11-5464/2016	India-1/Asia-5	MF964186.1
**Civet**	CP8 TH/2017	Asia-4	In this study

Supplementary Table S2. Averaged nucleotide compositions of CDV Asian strains

LINEAGE	A	T	C	G
**ASIA-1**	29	29	21	21
**ASIA-2**	31	28	21	20
**ASIA-3**	28	31	21	20
**ASIA-4**	29	30	21	20
**ASIA-5**	30	28	21	21
